# Structural Identification of the Pacemaker Cells and Expression of Hyperpolarization-Activated Cyclic Nucleotide-Gated (HCN) Channels in the Heart of the Wild Atlantic Cod, *Gadus morhua* (Linnaeus, 1758)

**DOI:** 10.3390/ijms22147539

**Published:** 2021-07-14

**Authors:** Gioele Capillo, Eugenia R. Lauriano, Jose M. Icardo, Prabhugouda Siriyappagouder, Michal Kuciel, Stelios Karapanagiotis, Giacomo Zaccone, Jorge M. O. Fernandes

**Affiliations:** 1 Department of Veterinary Sciences, Polo Universitario dell’Annunziata, University of Messina, 98168 Messina, Italy; gcapillo@unime.it; 2 Institute of Marine Biological Resources and Biotechnology—National Research Council (IRBIM, CNR), Spianata S. Raineri, 98122 Messina, Italy; 3 Department of Chemical, Biological, Pharmaceutical, and Environmental Sciences, University of Messina, 98168 Messina, Italy; elauriano@unime.it; 4 Department of Anatomy and Cell Biology, Poligono de Cazona, Faculty of Medicine, University of Cantabria, 39011 Santander, Spain; 5 Faculty of Biosciences and Aquaculture, Nord University, 8026 Bodø, Norway; prabhugouda.siriyappagouder@nord.no (P.S.); ste.karap@gmail.com (S.K.); 6 Poison Information Centre, Department of Toxicology and Environmental Disease, Faculty of Medicine, Jagiellonian University, Kopernika 15, 30-501 Cracow, Poland; michalkuciel@gmail.com

**Keywords:** cardiac pacemaker, hyperpolarization-activated cyclic nucleotide-gated (HCN) channels, intracardiac neurons, innervation, Atlantic cod

## Abstract

Hyperpolarization-activated cyclic nucleotide-gated (HCN) channels are proteins that contain highly conserved functional domains and sequence motifs that are correlated with their unique biophysical activities, to regulate cardiac pacemaker activity and synaptic transmission. These pacemaker proteins have been studied in mammalian species, but little is known now about their heart distribution in lower vertebrates and c-AMP modulation. Here, we characterized the pacemaker system in the heart of the wild Atlantic cod (*Gadus morhua*), with respect to primary pacemaker molecular markers. Special focus is given to the structural, ultrastructural and molecular characterization of the pacemaker domain, through the expression of HCN channel genes and the immunohistochemistry of HCN isoforms, including the location of intracardiac neurons that are adjacent to the sinoatrial region of the heart. Similarly to zebrafish and mammals, these neurons are immunoreactive to ChAT, VAChT and nNOS. It has been shown that cardiac pacemaking can be modulated by sympathetic and parasympathetic pathways, and the existence of intracardiac neurons projecting back to the central nervous system provide a plausible link between them.

## 1. Introduction

In contrast to the contractile organs in protostomes, the morphology of the heart in deuterostomes became more complex due to the higher demands on oxygen distribution. With the exception of the Cyclostomata, the adult fish heart is formed by six chambers, or segments, arranged in the following series: sinus venosus, atrium, atrioventricular canal, ventricle, conus arteriosus, and bulbus arteriosus. Several of these chambers have acquired different morphological and functional significance across the phyletic scale (see [1]). In teleosts, the two main cardiac chambers, ventricle and atrium, are distinguished by the expression of chamber-specific genes, regulating their establishment and maintenance [2]. Amniotes are characterized by the transition to a fully separated heart with four chambers, as found in crocodilians, avian, and mammals. Contractile myocytes have replaced mesothelial cells, and endocardial cells line the inner surface of the heart. Thus, in vertebrates, a variety of specialized cell types have replaced the cardiac mesothelium in the pumping organs in invertebrates. Excitation of the heart occurs in a specialized region known as sinoatrial node (SAN), which is essential for the maintenance of a normal heart rhythm. SAN is regulated by extrinsic (central nervous system) and intrinsic factors, such as the intracardiac neurons, natriuretic peptides, and mechanical forces [3]. In zebrafish, the primary pacemaker is found in a ring-like structure at the location of the sinoatrial valve [4,5,6]. However, in some fish species, no ultrastructural differences between the pacemaker tissue and myocardium were observed, except for a particular arrangement of the myocardium in some teleosts [7]. The criterion allowing a distinction of the nodal cells from other cardiac muscle cells in higher vertebrates, is their relative poorness in myofibrils within the cytoplasm. This characteristic is present in some parts of the sino-atrial myocardium of the loach [8], catfish [9], and trout [10], but more investigations are needed in order to draw a definite conclusion. Localization of myofibril-poor cells, as in the case of the Keith and Flack node, is not obvious in fish, but in the catfish, a scattering of nodal-like fibers is observed in the part that is closest to the sinus venosus, by anatomical and electrophysiological studies [11].

Hyperpolarization-activated cyclic nucleotide-gated (HCN) protein channels are a multigene family that play a role in establishing the pacemaker rate of the vertebrate hearts; this is unlike the hagfish, where the pacemaker activity and HCN expression reside in multiple areas of the atrium and ventricle of the aneural heart [12]. Recent electrophysiological studies, by [13], pointed to the pacemaker protein HCN4, and its correlation with pacemaker currents in the hearts of zebrafish. Also, Hassinen et al. (2017) have reported the expression of six HCN transcripts in brown trout (*Salmo trutta fario*) sinoatrial (SA) pacemaker cells, despite a small functional I_f_ current. This is in agreement with the anatomical identification of the pacemaker cells in the SAR (sinoatrial region) and AVR (atrioventricular region) of the zebrafish heart [6], where the population of Isl1-positive cells in the SAR completely overlapped the population of HCN4-IR cells, and also the primary initiation site of electrical activity from the SAR to AVR. In hagfish, its heart rate is set via pacemaker cells [14,15]. These cells are characterized by HCN protein channels in the cell membrane, which are responsible for slow depolarization of the membrane. In the heart and nervous system of mammals, HCN channels contribute to neuronal and cardiac firing rates. Further, [12] discovered six isoforms of HCN, equivalent to HCN 2, 3 and 4 of mammalian muscle HCN in hagfish. Their expression is reported in all cardiac chambers, but HCN3a is higher in atrial and ventricular muscle, suggesting that HCN4 dominance in adult mammalian hearts appeared after hagfish divergence.

The available data concerning intracardiac innervation in some fish species are those reported by [16] and [5,6], which emphasize the occurrence of the main localization of the nervous tissue in the sinoatrial plexus, which is regarded as the automatism (pacemaker) center of the heart. This plexus is a well-developed network of nerve fibers and nerve cell bodies (intracardiac neurons), concentrated in the SAR, but also distributed along the entire atrial canal up to the atrio-ventricular junction [5]. The sinoatrial region, known as sinoatrial node (SAN), is regulated by extrinsic sympathetic and parasympathetic innervation, and an intracardiac nervous system comprising the intracardiac neurons that are embedded in the myocardium. These neurons contain parasympathetic, and non-adrenergic and non-cholinergic (NANC) transmitters in fish hearts [16,17], and form intracardiac circuits that are important for the internal processing of extrinsic inputs and for intracardiac reflex control of cardiac function [3]. In fact, recent immunohistochemical evidence has demonstrated the expression of adrenergic beta 2 and cholinergic muscarinic type 2 receptors in the pacemakers of both the sinoatrial and atrioventricular regions in the zebrafish heart [6].

It is now well established that the teleost lineage experienced a specific whole-genome duplication that is thought to have occurred 320–350 million years ago, following their split from the holosteans [18]. Whole-genome duplication is a major force of adaptive genome evolution, since it generates duplicate genes that can be lost, undergo sub-functionalization (functional divergence), or acquire new functions (neofunctionalization) [19]. These additional paralogous genes add an extra layer of complexity to the molecular regulation of all biological processes, including cardiogenesis and pacemaker function [20]. To the best of our knowledge, the molecular characterization of HCN isoforms in teleost hearts is very limited. Phylogenetic analyses have indicated the existence of four HCN genes that are homologs to urochordate isoforms. Additional lineage-specific duplications appear to have evolved in urochordate and fish genomes [21].

In the present study, we examined the structural and immunohistochemical features of pacemaker cells, and their innervation in the heart of Atlantic cod (*Gadus morhua*), which is one of the best studied paracanthopterygians, due to their commercial importance. Moreover, we have integrated these data with the expression of four *hcn* paralogues in different areas of the Atlantic cod heart.

## 2. Results

### 2.1. Morphological Characterization of the Pacemaker in Atlantic Cod

**Figure 1:**
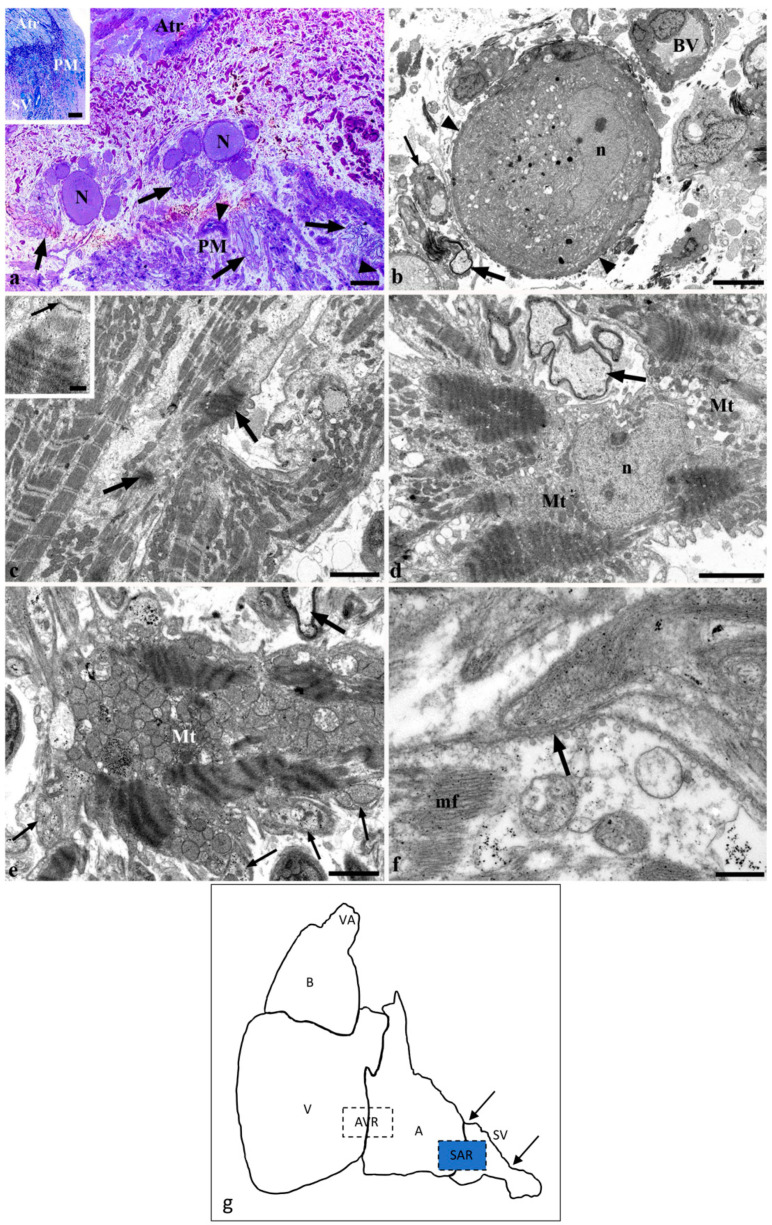
Histology and structure of the sinoatrial region and pacemaker area. (**a**) Semithin section. Sinus venosus wall. Large neuronal bodies (N) locate between the atrial myocardium (Atr) and the pacemaker (PM) area. The pacemaker tissue contains loosely organized myocardial bundles, small blood vessels (arrowheads) and abundant nerve bundles (arrows). Other nerve bundles locate close to the neuronal bodies. Inset of a: semithin section. Low magnification of the sinoatrial region. The atrium (Atr) and sinus venosus (SV) are separated by connective tissue. The pacemaker area (PM) is indicated. (**b**) TEM. A neuronal body showing nucleus (n) and nucleolus, lysosomal bodies and supporting glia (arrowheads) is surrounded by blood vessels (BV) and myelinated (thick arrow) and unmyelinated (thin arrow) nerve fibers. (**c**) TEM. The cytoplasm of the central myocyte shows two small areas of myofibrillar condensation (arrows). The surrounding cells show myofibrils with a regular appearance. Inset of **c**: TEM. Detail of myofibrillar condensation. The same myofibril shows normal Z band (arrow) and tightly packed myofilaments with condensed Z bands. (**d**) TEM. Myocardial cells show condensed myofibrils and regular mitochondria (Mt). Moreover, n is the myocardial nucleus; arrow is the myelinated nerve fiber. (**e**) TEM. A large cytoplasmic area contains condensed myofibrils surrounded by tightly packed rounded mitochondria (Mt). Note the presence of one myelinated (thick arrow) and several unmyelinated (thin arrows) nerve fibers. (**f**) TEM. One unmyelinated fiber appears in close apposition (arrow) with the myocardial cell surface. Specialized junctions are absent. mf indicates myofibrils. (**g**) Schematic drawing of the cardiac regions: VA, ventral aorta; B, bulbus arteriosus; V, ventricle; AVR, atrioventricular region; A, atrium; SAR, sinoatrial region; SV, sinus venosus. The area highlighted in blue indicates the region of observation. Scale bars: a: 50 µm; inset of a, 100 µm; b: 5 µm; c, 3 µm; inset of c, 500 nm; d, 3 µm; e, 2 µm; f, 500 nm.

The sinus venosus wall contained abundant collagen, fibroblasts, and discrete bundles of smooth muscle cells. The latter component became more apparent, and was better organized, near the atrium. In addition, the wall of the sinus venosus contained accumulations of neurons and numerous nerve bundles (Figure 1a). The neurons and nerves were surrounded by connective tissue, but were not encapsulated in any way (Figure 1a). The neurons showed a nucleolus, intranuclear rodlet, lysosomes in the vicinity of endoplasmic reticulum, and supporting glia (Figure 1b). The nerves in the sinus wall mostly contained myelinated fibers, but non-myelinated fibers were also present. Nerve bundles were distributed throughout the sinus venosus wall, but neuronal groups occurred more frequently in the sinoatrial region, close to the atrial myocardium and the pacemaker area (Figure 1a).

### 2.2. Differential Expression of Hcn Paralogues in Heart

**Figure 2:**
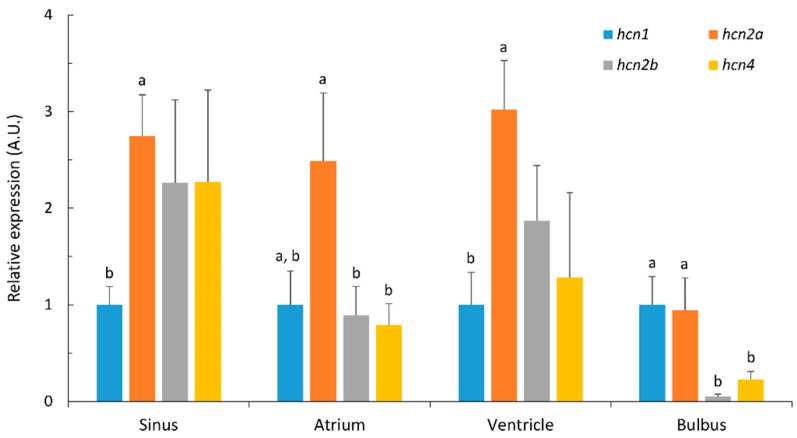
Relative expression levels of *hcn1*, *hcn2a*, *hcn2b* and *hcn4* in sinus, atrium, ventricle and bulbus arteriosus in Atlantic cod. Transcripts were quantified by qPCR, normalized using the geometric average of *ubi* and *eef1* expression and shown as relative values compared to *hcn1* transcript levels in each sample. Data are expressed in arbitrary units (A.U.) as mean ± S.E. (*n* = 5). Different superscript letters (^a, b^) indicate significant differences in transcript levels between *hcn* paralogues in each heart region. Differences in *hcn* transcript levels within each heart area were determined by one-way ANOVA with a Holm–Sidak post hoc test (*p* < 0.05). Kruskal–Wallis one-way ANOVA on ranks with Dunn’s post hoc test was performed when the data did not meet the normality and equal variance requirements.

All of the *hcn* paralogues that were examined (*hcn1*, *hcn2a*, *hcn2b* and *hcn4*) were ubiquitously expressed in the Atlantic cod heart, albeit at varying levels in different regions (Figure 2). In the sinus venosus, *hcn2a*, *hcn2b* and *hcn4* were the predominant paralogues, and were expressed at similar levels. There was a significant difference between the *hcn2a* and *hcn1* transcript levels, with *hcn2a* being 2.7-fold more abundant than *hcn1*. The most highly expressed paralogue in atrium was *hcn2a*, which was 2.8- and 3.1-fold higher than *hcn2b* and *hcn4*, respectively. It was also 2.5 times more abundant than *hcn1*, but this difference was not statistically significant. *Hcn2a* was also the predominantly expressed paralogue in the ventricle, and its transcript levels were three-fold higher than *hcn1* transcripts. In the bulbus arteriosus, *hcn1* and *hcn2a* were expressed at similar levels, and their transcripts were significantly more abundant than *hcn2b* and *hcn4*. *Hcn1* mRNA levels were 20.7- and 4.4-fold higher than *hcn2b* and *hcn4*, respectively. Similarly, *hcn2a* transcripts were 19.6- and 4.2-fold more abundant than *hcn2b* and *hcn4*, respectively.

### 2.3. Overview of the Innervation Pattern

**Figure 3:**
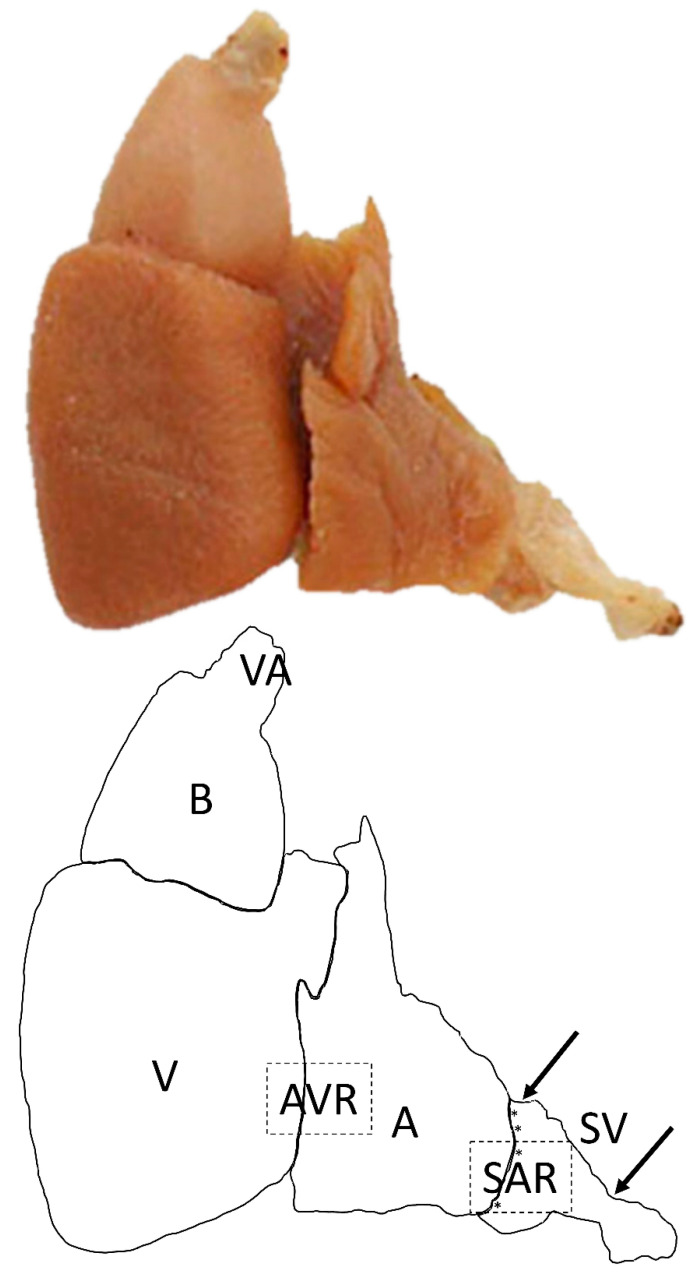
The heart of Atlantic cod (*Gadus morhua*) as it appears after removal from the body. Schematic indicates the cardiac regions: VA, ventral aorta; BA, bulbus arteriosus; V, ventricle; AVR, atrioventricular region; A, atrium; SAR, sinoatrial region; asterisks indicate the region of the sinoatrial plexus (SAP); SV, sinus venosus.

A schematic of the cod heart showing disposition of various chambers is reported in the Figure 3.

**Figure 4:**
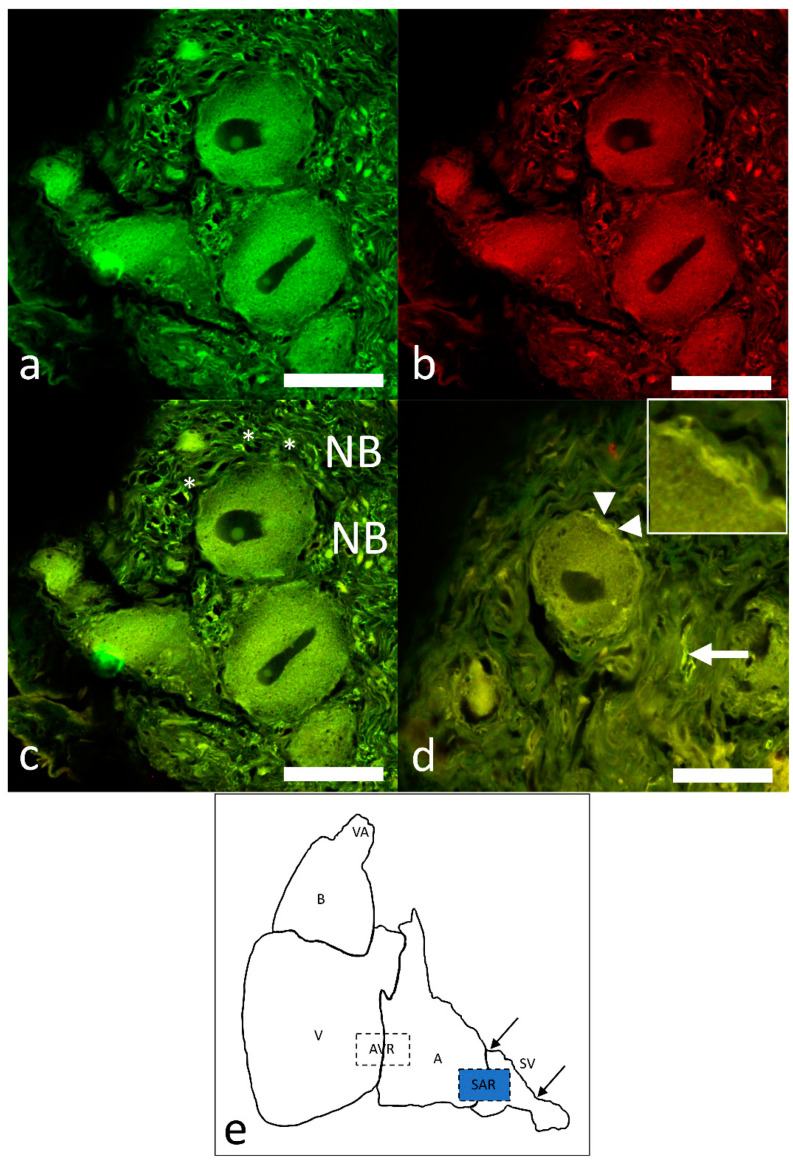
(**a**–**c**). Confocal images of intracardiac neurons immunostained with antibodies against ChAT and nNOS in the SAR. (**a**) (ChAT, green), (**b**) (nNOS, red) and (**c**) (merge) showing a complete overlapping of ChAT- and nNOS-positive neurons. Note the nerve fibers forming thick bundles (NB) are present in close vicinity of the presumptive pacemaker tissue, which is indicated by asterisks. (**d**) ChAT–nNOS neural terminals that surround the neuron and contain varicosities (arrowheads and inset). (**e**) Schematic indicates the cardiac regions: VA, ventral aorta; BA, bulbus arteriosus; V, ventricle; AVR, atrioventricular region; A, atrium; SAR, sinoatrial region; SV, sinus venosus. The blue highlighted area indicates the region of tissue sampling. Scale bars: 20 µm.

The intracardiac nervous system was demonstrated with the pan-neuronal markers AcT and Hu, showing the innervation of the sinoatrial region (SAR) of the heart of this species. A nerve plexus, the sinoatrial plexus (SAP), is located at the venous pole of the heart. AcT and Hu labeling revealed that axons coursed within the sinoatrial plexus and the somata of the intracardiac neurons (ICNs). Immunolabeling revealed the pattern of the SAR innervation, but did not indicate a differentiation between the axons originating from the extracardiac neurons and those arising from neurons, with their somata located intracardially (ICNs). Double immunolabeling with antibodies against ChAT and nNOS revealed a complete co-localization of the two neuronal markers (Figure 4a–d). Varicose nerve fibers that were double labelled with these antibodies were seen in close association at the surface of ChAT–nNOS–immunopositive neuronal somata (Figure 4d).

**Figure 5:**
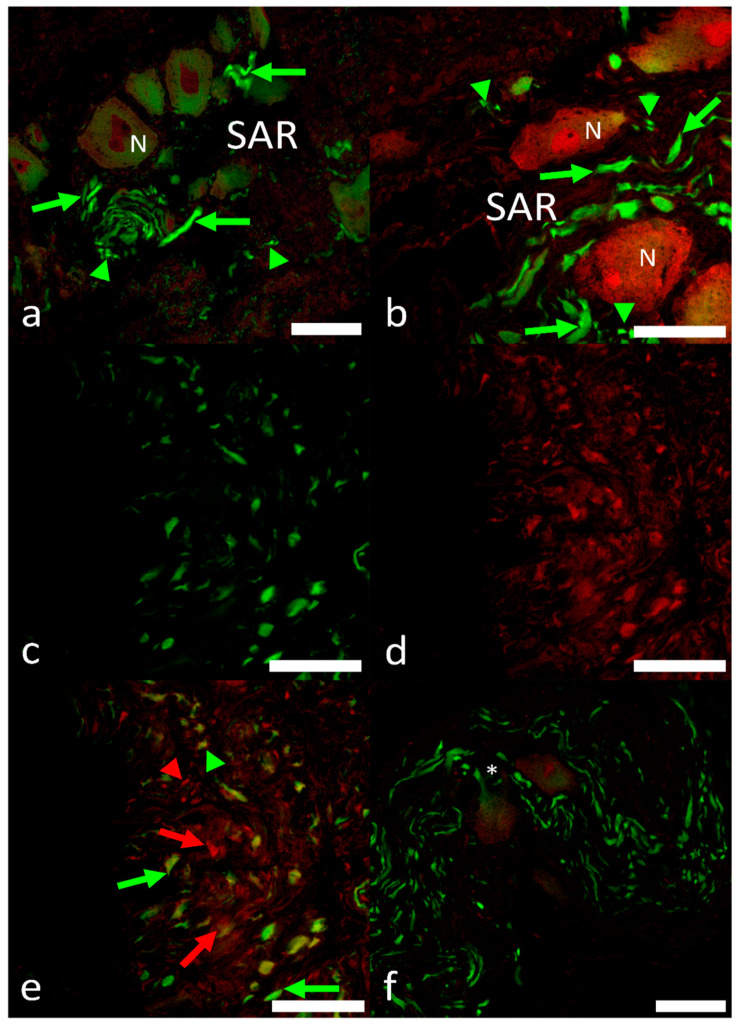
(**a**,**b**) Simultaneous detection of AcT and VAChT revealing abundant axon profiles (arrows) and nerve terminals (arrowheads) within the sinoatrial region (SAR). Cholinergic terminals (arrowheads) are identified among the neuronal somata (N). (**c**–**e**) A dense meshwork of both AcT and VAChT (c, d AcT and VAChT, respectively) axons and cholinergic terminals (e; merged) in the SAR is seen. (**f**) Neuronal somata in the AcT–immunoreactive axons neural meshwork located in the SAR. An asterisk indicates the axon from the hillock in a neuronal cell body. Scale bars: 20 µm.

Intense ChAT immunostaining was observed in axonal varicosities interconnecting the neuronal somata. The simultaneous detection of AcT and VAChT demonstrated two distinct populations of neuronal somata (neuron subtypes). A higher number of axon profiles and nerve terminals is noticed in the SAR (Figure 5a,c,d,f). Sometimes AcT-positive large axons, at single pole of soma originating from hillock, were observed (Figure 5f). Most of the clustered neuronal somata are positive to VAChT, and AcT-positive axons appeared to surround these somata (Figure 5f).

Sometimes AcT-positive terminals were apposed to VAChT-positive neuronal somata. In both cases, the axon profiles form varicosities “en passant” [22]. The axons terminals within the SAR were not double labeled with AcT and VAChT antibodies, thus indicating separate nerve fiber populations. Few axons exhibited co-labelling of the neuronal markers. Varicose AcT, VAChT and ChAT-nNOS axons were observed in close association with cardiomyocytes in the SAR and the atrium.

### 2.4. Identification of Pacemaker Cells

**Figure 6:**
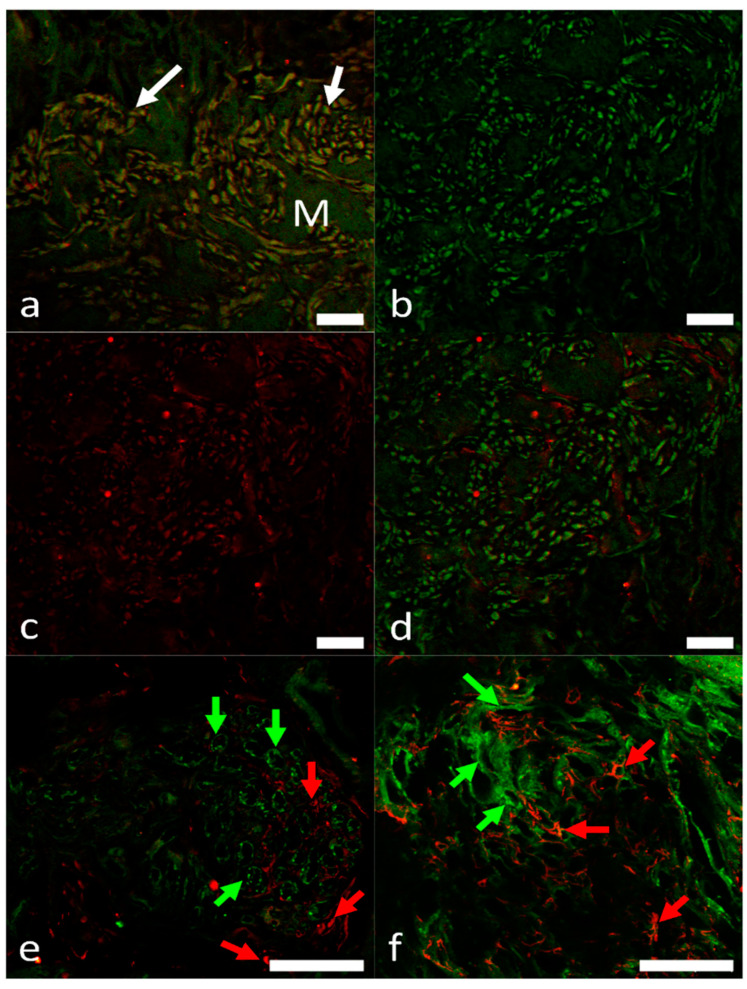
Multiple clusters of pacemaker cells detected by the double immunolabeling with antibodies against Islet-1 (green signal), and HCN1 and HCN2 (red signal) in the SAR. (**a**) Pacemaker cells (arrows) embedded in the myocardium (M) expressing Islet-1 (green signal) and HCN1 (red signal). (**b**–**f**) Pacemaker cells revealed colocalization of Islet-1 and HCN2 (**d**; merge). Two populations expressing Islet-1 (green signal and arrows) and HCN2 (red signal and arrows), respectively, were observed in e and f. Scale bars: 20 µm.

In the SAR region, antibodies to HCN isoforms (HCN1, HCN2, HCN4) were used to distinguish pacemaker cells from the surrounding cardiomyocytes. Cells expressing HCN1, HCN2 and HCN4 formed clusters that were intermingled with myocardial cells. HCN immunoreactivity is localized to either the cytoplasm or the cell membrane. Colocalization studies showed that most of the isoforms were often co-expressed with Islet-1 (Figure 6a,d). However, the combination of the antibodies to Islet-1, HCN1 and HCN2 revealed the presence of distinct cell populations, containing immunoreactivities to single markers (Figure 6e,f), probably due to cell size, ionic current densities, or a response to autonomic modulation [23,24].

**Figure 7:**
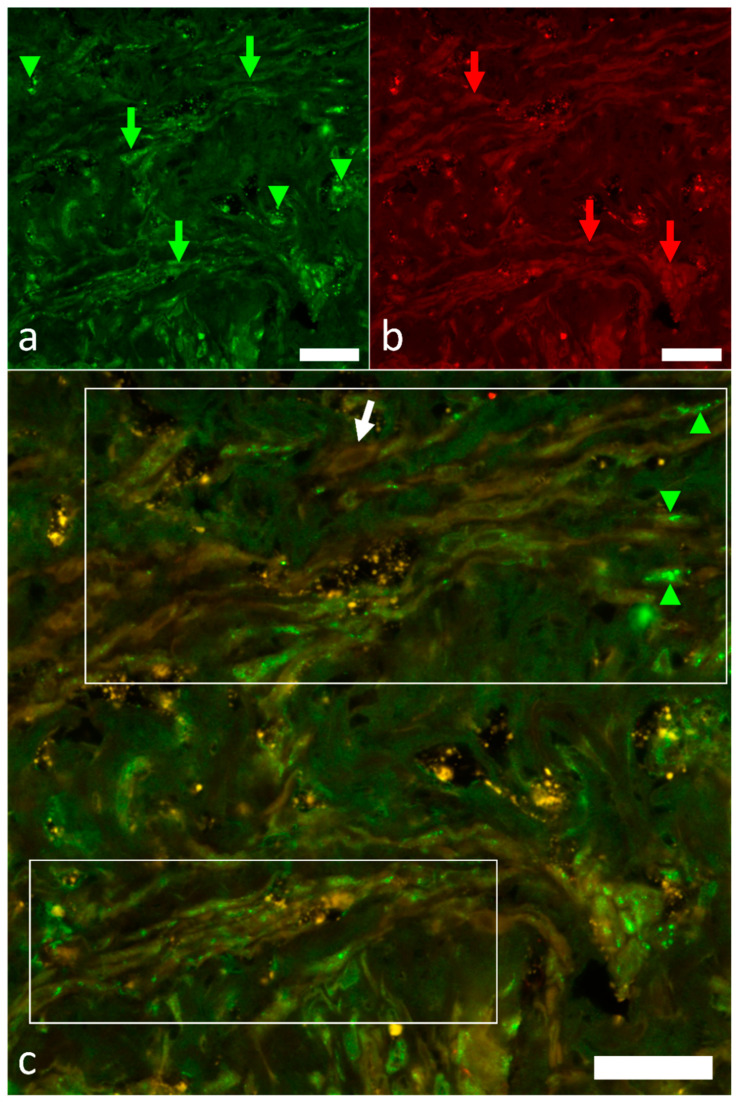
Double immunolabeling with antibodies against Hu ((**a**), green signal and arrows) and HCN4 ((**b**), red signal and arrows) in the SAR. HCN4 was weakly expressed in the pacemaker cells (green, red, and white arrows) that appeared to be innervated by Hu-immunoreactive axons and nerve fibers (arrowheads). Areas of pacemaker tissue with abundant pacemaker cells are boxed in (**c**). Scale bars: 20 µm.

The simultaneous detection of HCN4 and Hu has been used to identify the presence of the pacemaker cells among Hu-immunoreactive nerve fibers and axon bundles (Figure 7a–c).

Double immunolabeling with ChAT and nNOS antibodies revealed that numerous and sparse thick axon bundles are seen adjacent to the clustering of neuronal somata and the presumptive pacemaker region (Figure 4a–c).

## 3. Discussion

We have shown that the wall of the sinus venosus in *G. morhua* contains a complex pattern of nerves and neurons. Most of the cardiac innervation in teleosts is conveyed by vagosympathetic trunks that enter the heart following the sinus venosus wall (for a recent review, see Icardo, 2017). The cardiac output is regulated by the parasympathetic limb that causes cardio inhibition, and sympathetic limb that causes cardioexcitation [5,16,25]. Early and late studies have indicated that these nerves interact with resident intracardiac neurons, establishing a rich nervous plexus at the sinoatrial junction [5,22,26,27]. More than 90% of these neurons have been characterized as postganglionic cholinergic [5], although the final rate may vary between species [27,28]. In our study, we have demonstrated that ICNs in the SAR showed expression ChAT, VAChT and nNOS, and thus were cholinergic and nitrergic neurons that may innervate the pacemaker tissue. Also, preganglionic axons, presumably originating from vagosympathetic trunks, were positive for ChAT and nNOS, and were seen in close contact with ICNs, thus suggesting that Ach is released at the preganglionic synaptic junctions, acting on nicotinic receptors [5]. The presence of nNOS/NO in these terminals should also be correlated with its influence on afferent neurons and circuitry neurons in the heart.

In zebrafish, the sinoatrial plexus also contains efferent adrenergic neurons, and even afferent neurons projecting centripetally [5]. In the same sinoatrial junctional area, structural, immunological and electrophysiological studies have localized the heart pacemaker [4,5,26,27,29,30]. Colocalization studies showed that HU-positive nerve terminals and axon bundles approached HCN4 pacemaker cells, thus indicating the innervation of the pacemaker region by extrinsic and intrinsic nerves [22]. Our results in Atlantic cod are in agreement with previous findings, indicating a common pattern in the teleost heart.

From a structural point of view, the pacemaker area is located in the immediate vicinity of the atrial musculature, being surrounded by connective tissue. This area is not bound by any connective capsule and differs from the atrial myocardium, by having wide intercellular spaces, vascular profiles, and abundant axon terminals that establish numerous contacts with the surface of the myocardial cells. Previous structural studies have tentatively identified the pacemaker area on the basis of the abundance of neuromuscular junctions, and have focused on the richness and structure of the nerve terminals [22,26,29]. Curiously, the structure of the myocardium appears to have been mostly overlooked, except for a report indicating the absence of atrial specific granules (i.e., those containing the atrial natriuretic peptides) [29], and for several comments on the paucity of the myofibrillar content. This is in line with the poor myofibrillar content of the atrial myocytes (see [31]). The present results indicate that many myocardial cells in the pacemaker area show total or partial myofibrillar distortions, due to myofilament condensation, close apposition of the Z band-like material and disappearance of the other myofibrillar bands. Of note, single myofibrils may alternate condensed areas with areas showing the normal cross-striated pattern. The presence of “normal” myocardial cells and of cells with myofibrillar condensation could indicate the existence of cell subpopulations within the pacemaker area. This is in line with our colocalization studies, which showed that Islet-1 was not co-expressed with the HCN1 and HCN2 isoforms, and at least two cell subpopulations are present. The abundance of mitochondria, the lateral aggregation of myofilaments, and the accumulation of Z bands may be interpreted as images of nascent myofibrils, suggesting that these cells are not fully differentiated. Thus, they may retain embryological characteristics, such as the capability for spontaneous depolarization at a high rate. This suggestion is compatible with the expression of the transcription factor Isl-1 that is up-regulated in the embryonic myocardium, being later restricted to the sinus node in mammals [32], and to the sinoatrial junction in adult zebrafish [4] and goldfish [27]. Of note, HCN4- and Isl-1-positive myocardial cells in the sinoatrial valve of the goldfish coexist with myocardial cells that are negative to both HCN4 and Isl-1 [27].

In the nervous system and the heart of mammals, HCN channels contribute to the regulation of neuronal and cardiac firing rates. The currents produced by HCN channels are classified into Ih (hyperpolarization), Iq (queer), or If (funny) currents. Especially, Ih control the rhythmic activity of cardiac pacemaker cells, and spontaneous firing of neurons. Highly conserved sequences among the HCN channel family indicate that the vertebrate HCN1-4 genes arose from duplication of a single ancestral gene, prior to the lineage divergence. The functional properties of HCN1-4 channels are similar, but not identical. Functional differences among these paralogues may be predominantly due to motifs in their intracellular N- and C- termini [14,33].

In Atlantic cod cardiac myocytes, paralogues of *hcn1*, *hcn2a*, *hcn2b* and *hcn4* are expressed at varying levels in different cardiac regions, but paralogues to *hcn2a*, *hcn2b* and *hcn4* appeared to be expressed at the same levels in the sinus venosus, with a maximal expression of *hcn2a* in the sinus, atrium and ventricle. On the other hand, *hcn1* and *hcn2a* were the major channel proteins that were expressed in the bulbus arteriosus. Orthologs of all four mammalian *hcn* genes (hcn1–4) were expressed in brown trout heart [14,34]. The two paralogues of *hcn2* in Atlantic cod and the six in trout arose from duplications of *hcn* genes in the fish lineages [14]. There is a difference in cardiac *hcn* channel composition among Atlantic cod, brown trout, and mammalian heart. *Hcn4* is the predominant isoform of the *hcn* transcripts in rabbit and murine SA nodes [35]. The major expression of *hcn3* orthologs in brown trout is strikingly different to the mammalian heart [14]. HCN4 proteins were reported in the cardiac pacemaker tissue of the adult zebrafish and goldfish [27]. Notably, the pacemaker tissue of the trout contains both c-AMP (cyclic adenosine monophosphate)-insensitive (*hcn3* and *hcn1*) and c-AMP-sensitive (*hcn4* and *hcn2a*) isoforms, while in the atrium and ventricle, there are c-AMP-insensitive isoforms [14]. The analyses of molecular data revealed the presence, in the cod pacemaker tissue, of c-AMP-sensitive *hcn-2* isoforms that are also mostly expressed in the atrium and ventricle. Unlike HCN3, HCN4 and HCN2 are strongly dependent on c-AMP levels [35]. In conclusion, it seems that the HCN channels in Atlantic cod are not similar to those in the trout and hagfish [12,14]. The physiological significance of these differences awaits investigation, as well as the specific contribution of c-AMP modulation for the distinct and ubiquitous member of HCN2 in the sinus, atrium, ventricle, and bulbus arteriosus of the cod, since c-AMP is a crucial factor regulating Ih/HCN channel function in the heart and brain [36].

In the present study, we have not focused on the anatomical localization of a presumptive pacemaker tissue in other parts of the heart, such as the atrioventricular region. In the bulbus arteriosus, our molecular investigations proved the presence of a higher expression of *hcn2*. HCNs were commonly associated with both central and peripheral nervous systems [37,38]. The bulbus arteriosus of fish hearts showed a coronary circulation that is regulated by autonomic nerves [39].

In conclusion, despite the vast morphological differences between the simple fish heart and the structurally more complex mammalian heart, there is a striking degree of evolutionary conservation of the fundamental and molecular pathways [20].

## 4. Materials and Methods

### 4.1. Collection of Samples

Wild Atlantic cod (*Gadus morhua*) (*n* = 12) were caught by line fishing in Saltfjorden (latitude: 67°16′32″ N and longitude: 14°33′26″ E) near Mørkvedbukta research station (Nord University, Bodø, Norway). The sex, length and weight of each fish were recorded (Tables S1 and S2). Fish were humanely sacrificed by a blow to the head with a priest, followed by transection of the spinal cord. Their heart was excised and carefully dissected in different regions (sinus venosus, atrium, ventricle and bulbus arteriosus), which were collected separately in cryotubes, immediately frozen using dry ice and stored at −80 °C until RNA extraction. For transmission electron microscopy (TEM), whole hearts from 5 individuals were fixed in 25 mL fixative consisting of 3% (*v*/*v*) glutaraldehyde (Sigma-Aldrich, Steinheim, Germany) and 0.5% (*w*/*v*) CaCl_2_ (Sigma-Aldrich) in phosphate-buffered saline (Sigma-Aldrich).

### 4.2. Immunohistochemistry

#### 4.2.1. General and Neurotransmitter-Specific Labeling

The immunohistochemical procedures in the present study were similar to those described previously regarding the neuroanatomical studies on the visceral organs in fishes [16,40,41,42]. Tissue sections (6 µm) were rinsed in PBS and transferred to a PBS solution containing 2% Triton X-100 (X-100), Sigma-Aldrich), 1% (*w*/*v*) bovine serum albumin (BSA, A9576, Sigma-Aldrich), and 1% (*v*/*v*) normal goat serum (NGS, G9023, Sigma-Aldrich) for 48 h at 4 °C with agitation; they were then incubated with primary antibodies (Table 1). Primary antibodies were diluted in a solution containing 0.25% Triton X-100, 1% BSA, and 1% NGS in PBS and incubated overnight, then transferred to a solution of PBS containing the appropriate secondary antibody conjugated to AlexaFluor 488 or 555 fluorophores (Life Technologies, Burlington, Canada). The incubation time with secondary antibodies was 1 h. Final rinsing of tissue sections was performed in PBS before mounting with Vectashield (Vector Laboratories Inc., Burlingame, USA) to reduce fluorophore photobleaching.

#### 4.2.2. Primary Antibodies and Antibody Specificity

The primary antibodies used in this study have been previously validated in zebrafish, bichir and gar hearts and mudskipper gill [5,6,17,40,43]. In the present study to determine the general innervation of the heart, we have used antibodies against acetylated tubulin (AcT), the human neuronal protein C/D (Hu). The anti-AcT and anti-Hu antibodies were considered the most appropriate antibodies in mammals with which to obtain reliable estimates of total number of enteric neurons [44]. Antibodies to AcT–Hu were also used for the description of zebrafish intracardiac neurons [6] and enteric neurons [45]. Hu proteins are involved in many posttranscriptional mechanisms for the development and maintenance of the nervous system.

Cholinergic axons and somata were detected by immunoreactivity for choline acetyltransferase (ChAT), an enzyme involved in Ach synthesis, combined with the antibody to neuronal nitric synthase (nNOS) to label the intracardiac innervation. In more specimens antibody against vesicular acetylcholine transporter (VAChT) was used together with ChAT to double label cholinergic elements, and with AcT to differentiate between cholinergic elements, neuronal fibers and neuronal cell bodies [44]. VAChT antibodies have been used to reveal the expression of acetylcholine in the neuroepithelial cells and the neurons in the fish gill [40,41]. Neurons capable of producing nitric oxide (NO) were detected by the presence of nNOS (monoclonal anti-nNOS and polyclonal anti-nNOS). The characterization, specificity and reliability of the antibodies directed against choline acetyltransferase (ChAT) and nNOS, and their application in morphological studies of the intracardiac ganglia containing a heterogeneous population of neurons [46], the zebrafish enteric nervous system [44] and the neuroepithelial cell system of the fish gill [41,47], have been previously reported.

#### 4.2.3. Pacemaker Cell Immunohistochemistry

Paraffin sections containing the sinoatrial region were treated with antibodies against HCN1, HCN2, HCN4, and the transcription factor Islet-1, to detect the pacemaker cells. Some specimen subsets were labeled using antibodies against all three HCN isoforms and the Hu neuronal protein to detect the innervation of the pacemaker cells. Pre-absorption of the primary antisera to HCN1, HCN2 and HCN4 with the respective blocking peptide (HCN1 blocking peptide, BLP-PC056, HCN2 blocking peptide, BLP-P030 and HCN4 blocking peptide, BLP-PCO52 from Alomone Labs) according to the guidelines of the supplier led to the complete elimination of the immunostaining of the pacemaker tissue. HCN4 antibodies as reliable markers of the pacemaker cells as well as the controls of HCN4 and Islet-1 antibodies in the heart of zebrafish were reported by [4,5].

#### 4.2.4. Analysis and Imaging

Processed specimens were viewed using a LSM 700 Zeiss confocal microscope. The sections were analyzed, and images acquired using a Zeiss LSMDUO confocal laser scanning microscope with META module (Carl Zeiss, Micro Imaging, GmbH, Jena, Germany). Zen 2011 (LSM 700, Zeiss software) built in colocalization view was used to highlight the expression of both antibody signals in order to produce a co-labeling signal. Digital images were cropped, and the figure montage was prepared by the use of Adobe Photoshop 7.0 (Adobe System, San Jose, CA, USA).

### 4.3. Semi-Thin Sections and Transmission Electron Microscopy (TEM)

Selected samples from the tissues fixed in 3% (*v*/*v*) glutaraldehyde were postfixed in 1% (*w/v*) osmium tetroxide, dehydrated in graded acetone and propylene oxide, and embedded in Araldite (Fluka, Buchs, Switzerland), following routine procedures. Semithin sections were cut with an LKB III ultratome, stained with 1% (*w*/*v*) toluidine blue, and observed with a Zeiss Axioskop 2 plus microscope equipped with an AxioCam HRc digital camera. For TEM, ultrathin sections cut with a Leica ultracut UCT were stained with uranyl acetate and lead citrate, and examined with a Jeol-JEM-1011 working at 80 KV and equipped with a Gatan ORISUS SC 1000 CCD camera.

### 4.4. RNA Extraction and cDNA Synthesis

Total RNA from the different heart segments was extracted following the QiAzol protocol (Qiagen, Germany). Purity and quantity of RNA were determined using the NanoDrop 1000 (Thermo Fisher Scientific, Waltham, MA, USA). RNA integrity was assessed by gel electrophoresis on 1.2% (*w*/*v*) agarose gels (Sigma-Aldrich) stained with SYBR safe DNA gel stain (Thermo Fisher Scientific). One microgram of total RNA from each sample was reverse transcribed using the reverse QuantiTect transcription kit (Qiagen), following the manufacturer’s protocol.

### 4.5. Real-Time PCR (qPCR) and Quantification of Gene Expression

For each heart region, the 5 samples with the best RNA quality were selected for qPCR analysis, as detailed in Supplementary Materials Table S1. Real-time PCR amplification was performed on a LightCycler^®^ 96 thermocycler (Roche Diagnostics, Basel, Switzerland). All PCR reactions were carried out in duplicate in 10 μL reactions consisting of 5 μL of LightCycler 480 SYBR green I master mix (Roche), 1 μL of gene-specific primer pair (5 μM each) and 4 μL of 25× diluted cDNA sample. Non-reverse transcription control and no template controls were included for each primer pair. Details of the primers used for target and reference genes are provided on Table 2. Thermocycling parameters were as follows: initial denaturation at 95 °C for 10 min, followed by 45 cycles at 95 °C for 10 s, optimized annealing temperature (58–65 °C) for each gene (Table 2) for 30 s and 72 °C for 20 s. The specificity of amplification was determined by melting curve analysis. The standard curve was obtained by running a 5-point series of 2-fold dilution (1:2, 1:4, 1:8, 1:16 and 1:32) pooled cDNA. Relative expression of the target genes was determined using GeNorm [48], as reported in [49]. The geometric means of the two most stable reference genes (*eef1a* and *ubi*) were used as calibrators. Differences in *hcn* transcript levels in each heart area were determined by one-way ANOVA with a Holm–Sidak post hoc test (*p* < 0.05). Kruskal–Wallis one-way ANOVA on ranks with Dunn’s post hoc test was performed when the data did not meet the normality and equal variance requirements. Statistical analyses were performed with the SigmaStat statistical package (Systat software, UK).

## Figures and Tables

**Table 1:** Primary and secondary antibodies used in the present study.

Primary Antibody	Clonality	Manufacturer	Catalogue Number	Dilution	ID Number
Monoclonal anti-tubulin, acetylated antibody (AcT)	Monoclonal	Sigma-Aldrich	T6793	1:1000	AB_477585
Mouse anti-human HuC/HuD neuronal protein (Hu)	Monoclonal	Molecular Probes	A21271	1:250	AB_221448
Choline acetyltransferase (ChAT)	Polyclonal	Atlas Antibodies	HPA048547	1:50	AB_2680437
Anti-vesicular acetylcholine transporter (VAChT)	Monoclonal	Millipore	AB1588	1:100	AB_2187981
Anti-vesicular acetylcholine transporter (VAChT)	Polyclonal	Sigma-Aldrich	V5387	1:500	AB_261875
Neuronal nitric oxide synthase (nNOS)	Monoclonal	Abcam	ab67002	1:25	AB_1141642
Neuronal nitric oxide synthase (nNOS)	Polyclonal	Santa Cruz Biotechnology	sc-648	1:500	AB_630935
Anti-HCN1	Polyclonal	Alomone Labs	APC-056	1:100	AB_2039900
Anti-HCN2	Polyclonal	Alomone Labs	APC-030	1:100	AB_2313726
Anti-HCN4	Polyclonal	Alomone Labs	APC-052	1:100	AB_2039906
Islet-1	Monoclonal	DSHB	39.4D5	1:100	AB_2314683

**Table 2:** Details of the primers used for relative quantification of mRNA levels by qPCR. In addition to the primer sequences used to quantify each transcript level, amplicon sizes, annealing temperatures (Ta) and amplification efficiencies (E) are indicated.

Gene	Primer Sequence (5′–3′)	Amplicon Size (bp)	Ta (°C)	E (%)	Accession or Reference
*hcn1*	AGCGATTTTAGGTTCTACTGGG GGAAGATGGTGTCCGAGGC	146	58	82	XM_030342287.1
*hcn2a*	GACGTGAGGCAGAAGATCCA GTCGCTCAGCTCTCCCAGG	90	62	72	XM_030370466.1
*hcn2b*	ACAGTGACTTCAGCAGGTTCTAC AAGTTGAGCACCAGGTCCAT	173	62	78	ENSGMOG00000011849
*hcn4*	GTGATTTCAGGTTCTACTGGGAC CAGGTCCAGGAGGAAGAAGG	155	62	75	ENSGMOG00000001132
*arp*	ACGCACCAGCCAAGGTAGAG ATGTCGTCATCAGACTCCTCGG	70	60	89	EX741373.1
*eef1a*	ATCGGCGGTATTGGAACAGT CATCTCAACGGACTTCACCTCA	117	65	99	EX721840.1
*ubi*	GGCCGCAAAGATGCAGAT CTGGGCTCGACCTCAAGAGT	69	60	96	[50]

## Data Availability

The data presented in this study are available on request from the corresponding author.

## Supplementary Materials

The following are available online at https://www.mdpi.com/article/10.3390/ijms22147539/s1, Table S1: sex, length and weight of Atlantic cod used for gene expression analysis, Table S2: details of Atlantic cod individuals whose hearts were fixed for transmission electron microscopy.
