# Supplementary material for: Structural Identification of the Pacemaker Cells and Expression of Hyperpolarization-Activated Cyclic Nucleotide-Gated (HCN) Channels in the Heart of the Wild Atlantic Cod, Gadus morhua (Linnaeus, 1758)
Source: Int J Mol Sci. 2021 Jul 14;22(14):7539. doi: 10.3390/ijms22147539 (PMC8307021; doi:10.3390/ijms22147539)
Supplement: Supplementary file 1 [file ijms-22-07539-s001.zip › ijms-1281060-supplementary.pdf]

Table S1. Sex, length and weight of Atlantic cod used for gene expression analysis.

Fish No	Sex	Weight (kg)	Total length (cm)	Standard length (cm)
1	Male	2.05	63.0	58.0
2	Male	0.60	35.0	32.0
3	Female	1.55	58.0	53.0
4	Female	1.20	51.0	47.0
5	Male	1.40	49.5	45.5
6	Male	1.95	64.5	59.5
7	Male	1.90	59.5	55.0

Footnote: For each heart region, five samples with the best RNA quality were selected for qPCR analysis. Atrium: fish no. 1, 2, 3, 5 and 6; Ventricle: fish no. 1,2, 3, 5 and 6 Sinus venosus: fish no. 1, 2, 3, 5, and 6; Bulbus arteriosus: fish no. 2, 3, 4, 5, and 7.

Supplementary Table S2. Details of Atlantic cod individuals whose hearts were fixed for transmission electron microscopy.

Fish No	Sex	Weight (kg)	Total length (cm)
8	Female	0.40	33.0
9	Male	1.8	63.0
10	Male	0.40	32.0
11	Male	1.5	51.0
12	Female	0.40	33.0
